# In Pancreatic Adenocarcinoma Alpha-Synuclein Increases and Marks Peri-Neural Infiltration

**DOI:** 10.3390/ijms23073775

**Published:** 2022-03-29

**Authors:** Matteo Bianchini, Maria Giambelluca, Maria Concetta Scavuzzo, Gregorio Di Franco, Simone Guadagni, Matteo Palmeri, Niccolò Furbetta, Desirée Gianardi, Aurelio Costa, Manuel Gentiluomo, Raffaele Gaeta, Luca Emanuele Pollina, Alfredo Falcone, Caterina Vivaldi, Giulio Di Candio, Francesca Biagioni, Carla Letizia Busceti, Paola Soldani, Stefano Puglisi-Allegra, Luca Morelli, Francesco Fornai

**Affiliations:** 1 General Surgery Unit, Department of Translational Research and New Technologies in Medicine and Surgery, University of Pisa, 56124 Pisa, Italy; bianchini.matteo@yahoo.it (M.B.); gregoriodifranco@gmail.com (G.D.F.); simone5c@virgilio.it (S.G.); palmeri.matteo@gmail.com (M.P.); n.furbetta@hotmail.it (N.F.); gianardi.d@gmail.com (D.G.); giuliodicandio@gmail.com (G.D.C.); 2 Human Anatomy, Department of Translational Research and New Technologies in Medicine and Surgery, University of Pisa, 56124 Pisa, Italy; maria.giambelluca@dmu.unipi.it (M.G.); mariaconcetta.scavuzzo@dmu.unipi.it (M.C.S.); paola.soldani@unipi.it (P.S.); 3 General Surgery Unit, ASL Toscana Nord Ovest Pontedera Hospital, 56025 Pontedera, Italy; aurelio.costa@uslnordovest.toscana.it; 4 Department of Biology, University of Pisa, 56124 Pisa, Italy; manuel.gentiluomo@biologia.unipi.it; 5 Division of Surgical Pathology, Department of Surgical, Medical, Molecular Pathology and Critical Area, University of Pisa, 56124 Pisa, Italy; raffaele.gaeta@med.unipi.it (R.G.); l.pollina@ao-pisa.toscana.it (L.E.P.); 6 Division of Medical Oncology, Department of Translational Research and New Technologies in Medicine and Surgery, University of Pisa, 56124 Pisa, Italy; alfredo.falcone@unipi.it (A.F.); caterinavivaldi@gmail.com (C.V.); 7 IRCCS Neuromed-Istituto Neurologico Mediterraneo, 86077 Pozzilli, Italy; francesca.biagioni@neuromed.it (F.B.); carla.busceti@neuromed.it (C.L.B.); stefano.puglisiallegra@neuromed.it (S.P.-A.); 8 EndoCAS (Center for Computer Assisted Surgery), University of Pisa, 56124 Pisa, Italy

**Keywords:** pancreatic ductal adenocarcinoma, α-synuclein, Western blotting, electron microscopy, neuroinvasion, electron-microscopy, ultrastructural stoichiometry

## Abstract

α-Synuclein (α-syn) is a protein involved in neuronal degeneration. However, the family of synucleins has recently been demonstrated to be involved in the mechanisms of oncogenesis by selectively accelerating cellular processes leading to cancer. Pancreatic ductal adenocarcinoma (PDAC) is one of the most lethal human cancers, with a specifically high neurotropism. The molecular bases of this biological behavior are currently poorly understood. Here, α-synuclein was analyzed concerning the protein expression in PDAC and the potential association with PDAC neurotropism. Tumor (PDAC) and extra-tumor (extra-PDAC) samples from 20 patients affected by PDAC following pancreatic resections were collected at the General Surgery Unit, University of Pisa. All patients were affected by moderately or poorly differentiated PDAC. The amount of α-syn was compared between tumor and extra-tumor specimen (sampled from non-affected neighboring pancreatic areas) by using in situ immuno-staining with peroxidase anti-α-syn immunohistochemistry, α-syn detection by using Western blotting, and electron microscopy by using α-syn-conjugated immuno-gold particles. All the methods consistently indicate that each PDAC sample possesses a higher amount of α-syn compared with extra-PDAC tissue. Moreover, the expression of α-syn was much higher in those PDAC samples from tumors with perineural infiltration compared with tumors without perineural infiltration.

## 1. Introduction

Synucleins (syns) are a family of small, soluble, highly conserved proteins, which consist of α-syn, β-syn, and γ-syn [1,2,3,4]. A considerable amount of native α-syn quickly misfolds and aggregates through five to six repeats of amino acid motif occurring toward the N terminal [5,6,7]. These repeats produce the formation of conserved amphipathic A2-helices also characteristic of apolipoproteins, which mediate aggregation and reversible binding to membrane phospholipids. Although various cell functions of α-syn remain to be established, the protein is known to be involved in some neurodegenerative disorders [7]. In fact, α-syn is a major component of Lewy bodies [8,9,10,11] in Parkinson’s disease (PD), and it is often detected in Alzheimer’s disease [10,12,13,14,15,16,17,18]. Data about the occurrence of the syns family in tumors mostly investigated γ-syn, which may be aberrantly expressed [19]. Several studies show that γ-syn is highly expressed in various types of cancer, such as breast, ovarian, and colorectal cancer, especially in advanced stages of the diseases [20]. The overexpression of γ-syn stimulates proliferation and induces the invasion and metastasis of breast cancer cells in in vitro assays as well as in animal models [21]. γ-Syn has also been shown to alter mitotic checkpoint controls, thus producing multi-nucleation as well as more rapid breast cancer cell growth [22,23]. The overexpression of γ-syn also interferes with drug-induced apoptosis in breast and ovarian cancer [24]. α-Syn and β-syn are described in some types of cancer; however, these data are scattered, and specific investigations about α-syn did not appear until recent years. Baseline α-syn expression is highly tissue-specific and is mostly restricted to brain areas within neuronal nuclei and presynaptic terminals [14]. The involvement of α-syn in tumors stems from recent finding of high amount of α-syn in melanoma [25]. These studies were followed by investigation at a pre-clinical level to question whether, despite a coincidental expression, α-syn may exert an active biological effect in melanoma cells. In fact, very recently, Shekoohi and colleagues demonstrated that suppressing α-syn expression inhibits melanoma cell growth [26]. This evidence shifts the role of α-syn from a mere epiphenomenon witnessing metabolic dysregulation in catecholamine-producing cells toward an active determinant in oncogenesis [26]. In fact, as reviewed by Ejma and colleagues [27], multiple substrates of the autophagy process are active determinants in oncogenesis, while being concomitantly involved in neurodegeneration. This is the case of PTEN-induced kinase 1 (PINK1), which may either be oncogenic or suppress tumor growth; similarly, the protein parkin modulates tumor growth. In keeping with autophagy, mutations in the protein/nucleic acid deglycase named DJ-1 are key in some neoplasms, such as melanoma, breast, lung, colorectal, uterine, and hepatocellular cancers. Again, autophagy-dependent mitochondrial dysfunction is massively involved in cancer development [27].

In line with this, the overexpression of α-syn in melanoma coexists with marked alterations in the autophagy pathway. Consistently, autophagy alterations are now hypothesized as both biomarkers and prognostic factors in melanoma [28,29].

Pancreatic cancer is now the fourth most common cause of cancer death worldwide. Patients with pancreatic ductal adenocarcinoma (PDAC) have a dismal prognosis, as the overall mean survival time is 6 months and the 5 year survival rate is less than 10% [30,31,32]. At the time of diagnosis, less than 20% of patients are eligible for surgical resection, while 80% of cases are too advanced due to regional infiltration or distant metastasis [33]. The poor prognosis of pancreatic cancer, even in cases eligible for surgical resection, is due to extensive local infiltration and early spreading via lymphatic and hematic pathways. In fact, even after surgical resection, pancreatic cancer is characterized by early local or distant recurrence. The spreading pattern of PDAC often recruits the peri-neural pathways, which is defined as peri-neural invasion (PNI). PNI occurs in 90–100% of pancreatic cancers, and it can be observed early in the disease course since it is also detected in the absence of lymphatic or hematic metastasis, and even when the tumor is smaller than 2 cm [34]. The occurrence of PNI is deleterious for the prognosis of PDAC patients [35,36,37,38]. Thus, a better insight into the mechanisms underlying neurotropism represents an important issue to understand the molecular mechanisms operating at the onset and spread of pancreatic cancer. This is supposed to foster and develop novel specific therapeutic strategies [38]. 

Previous studies have shown that γ-syn is slightly overexpressed in pancreatic cancer [39]; however, the expression of α-syn in PDAC has never been analyzed. This is very important considering that α-syn is removed by autophagy [40]. Since an alteration of autophagy machinery in PDAC occurs [41], it is expected that autophagy-dependent proteins may be altered consistently. Among these proteins, α-syn may be useful to improve diagnostic tools in PDAC. In fact, recent autophagy research in PDAC was seminal to reveal new biomarkers and targets for potential PDAC diagnosis and treatment [42]. In line with this, recent data indicate that other autophagy-dependent proteins, such as cellular and scrapie-like prion protein (PrPc and PrPsc, respectively), are increased in PDAC and other neural crest-derived tumors [43,44,45,46]. It is important to consider that PrPc promotes α-syn expression by interacting with the α-syn gene (SNCA) promoter [47,48], and it induces hyperphosphorylation of α-syn [49]. Thus, the present study aims to assess the expression of the autophagy substrate, α-syn in PDAC samples (already known to express a high amount of PrPc [43,44]) compared with extra-PDAC tissue. When this occurs, a potential correlation between specific PDAC phenotypes (such as neurotropism) and the expression of α-syn is calculated. To address these issues, the expression of α-syn in PDAC and extra-PDAC tissue samples was measured. α-Syn was identified and counted by using immunohistochemistry and immune-electron microscopy along with Western blotting.

## 2. Results

### 2.1. Patients

Data from patients diagnosed with PDAC, including grading and staging, are summarized in Table 1. We collected surgical specimens from 28 patients, of which 8 were suddenly excluded from the study due to inadequate sampling or PDAC misdiagnosis at frozen section. Thus, a total of 20 patients were finally included in the study; 9 of them (45%) were males and 11 (55%) females. The mean age was 71.9 ± 7.7 years (range 52–87). The pathology exam confirmed the presence of PDAC in all 20 cases. The grading of the pancreatic tumor was “moderately differentiated” in 17/20 cases (85%) and “poorly differentiated” in 3/20 (15%). The mean tumor size was 3.4 ± 0.3 cm (range 1.5–6.5 cm). The mean harvested lymph nodes were 32.1 ± 3.3 (range 14–62) with the presence of metastatic lymph nodes in 17/20 cases (85%) and a mean number of metastatic lymph nodes of 4.9 ± 0.8 (range 1–12). The presence of angioinvasion was reported in 2/20 cases (10%), while the presence of peri-neural infiltration was reported in 16/20 cases (80%).

### 2.2. Histochemistry of PDAC and Extra-PDAC Areas

**Figure 1:**
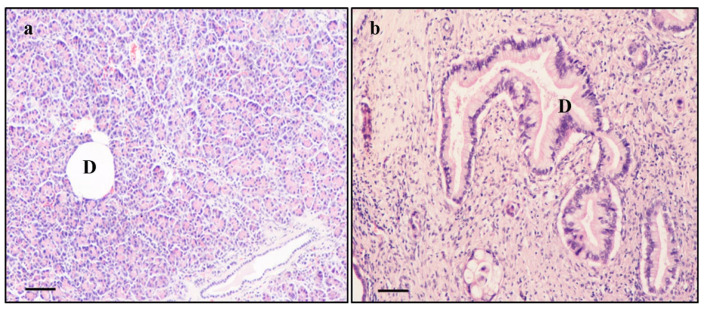
Histology of pancreatic tissue. Extra-PDAC (**a**) and PDAC, (**b**) following H & E histochemistry. Extra-PDAC tissue shows normal architecture with well-preserved ductal system (D). In contrast, PDAC tissue is composed of desmoplastic stroma in which the cells lose their integrity. The ducts (D) are enlarged and irregularly shaped. Scale bar = 25 µm.

Representative hematoxylin and eosin (H & E)-stained pictures from pancreatic extra-PDAC and PDAC tissue are shown in Figure 1. The histological organization of pancreatic extra-PDAC tissue possesses a normal exocrine acinar architecture with well-preserved ductal system, while the stroma of PDAC tumors is composed of abundant extracellular matrix with increasingly evident loss of cell architecture. The ducts are very enlarged and irregularly shaped. 

This plain H & E staining served as the guide to sample and validate tissue either from PDAC or extra-PDAC areas, which were further analyzed by using α-syn immuno-detection. When specimens were collected from a presumed PDAC, the extra-PDAC tissue was harvested at least 1 cm away. In this way, various areas in a single patient provided samples for measuring α-syn by Western blotting, to carry out α-syn immune-histochemistry, and to count α-syn particles by applying immuno-gold stoichiometry at electron microscopy. 

### 2.3. α-Syn Immuno-Histochemistry

**Figure 2:**
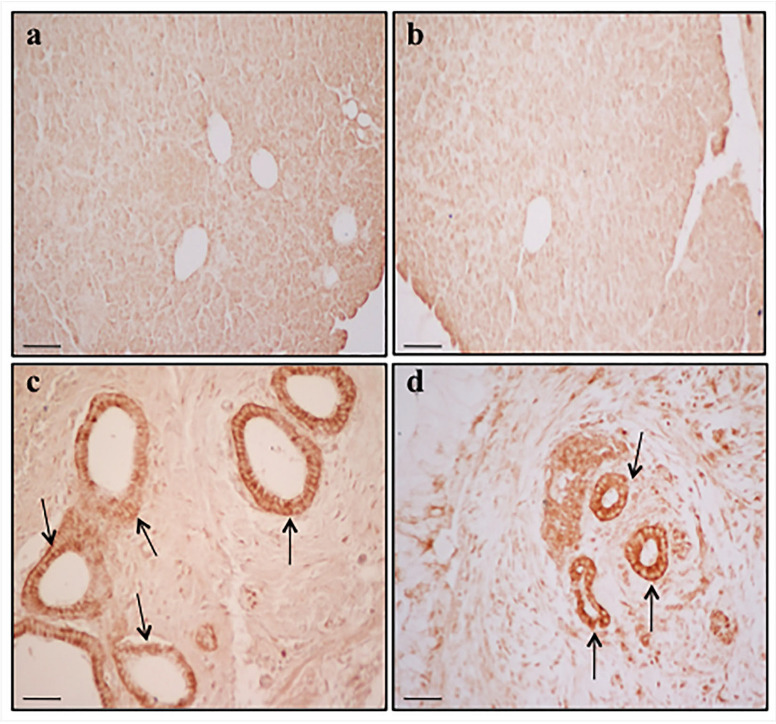
Expression of α-syn in pancreatic tissue in two patients. Immuno-peroxidase shows weak α-syn staining in extra-PDAC tissue (**a**,**b**). Immunoperoxidase shows α-syn-specific labelling in ductal cells (arrows) from PDAC samples (**c**,**d**). Anti-α-syn immuno-staining occurring in PDAC tissue is mainly focused in two areas, namely the ductal cells and some intense zones. These include stromal areas from PDAC, which possess intense α-syn immuno-staining according to patches and axon-like linear patterns (Figure 2). The identification of “intense zones” was based on detecting those stromal areas, which were intensely stained by immuno-peroxidase following exposure to α-syn primary antibodies. Scale bar = 30 µm.

**Figure 3:**
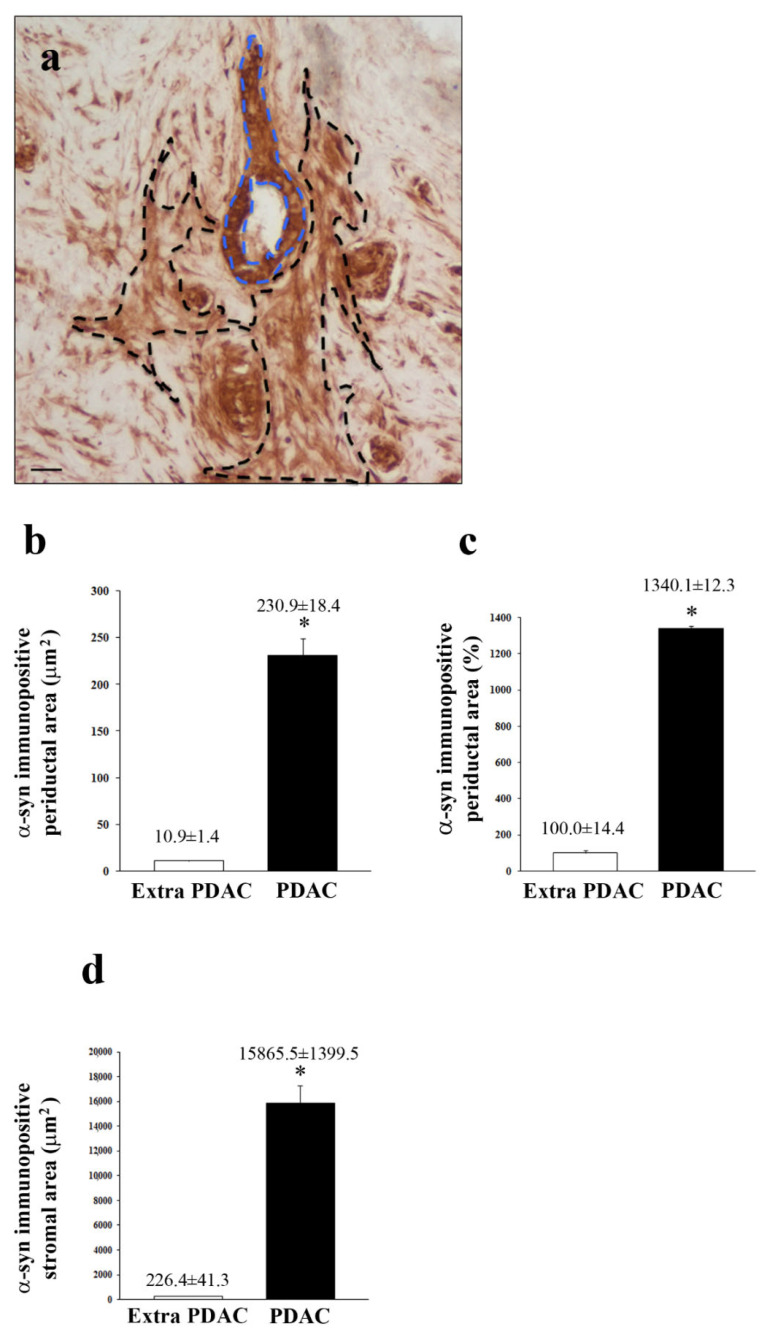
Measurement of α-syn immune-histochemistry. Representative picture from PDAC tissue (**a**). α-syn immune-staining is highly evident in both ductal and stromal regions. The dotted lines encircle the periductal (light blue) and stromal (black) areas as they are selected to carry out the measurement of α-syn immuno-stained areas. The counts of these areas are reported in the graphs. In detail, (**b**) measures the mean ± S.E.M. of α-syn immuno-stained ductal area given mm^2^ while (**c**) reports the percentage of α-syn immuno-stained ductal area in PDAC compared with extra-PDAC (extra-PDAC = 100). The difference between b and c is due to a larger ductal area in PDAC compared with extra-PDAC tissue, and it serves as a reference. (**d**) Stromal α-syn immuno-stained area is given in mm^2^. Values are given as the mean ± S.E.M. of *N* = 100 measures expressed either in surface units (mm^2^) or in percentage (**c**) mm^2^ per group. * *p* < 0.0001 (**b**–**d**). Scale bar = 30 µm.

α-Syn immuno-histochemistry of PDAC shows massive staining of the ductal cells, which is way in excess compared with the negligible immuno-staining of control extra- PDAC tissue (Figure 2). It is remarkable that the amount of a-syn immune-positive ductal cells were layered in PDAC. This phenomenon, joined with a larger ductal volume and consistent immunostaining of each ductal cell within the PDAC region, produces an enormous increase in a-syn immunostaining at the level of PDAC tissue compared with extra-PDAC specimen. In fact, when measuring immune-positive areas with light microscopy, the difference between PDAC and extra-PDAC regions largely surpasses what is detected by Western blotting. The stromal areas from PDAC possess intense zones of a-syn immuno-staining according to patches and axon-like linear patterns (Figure 2). The identification of “intense zones” was based on detecting those stromal areas pancreatic tissue, which were intensely stained at immuno-peroxidase following exposure to a-syn primary antibodies. The pattern of a-immuno-staining explains the marked differences between the PDAC and extra-PDAC regions when they were measured according to Figure 3a. In fact, as shown by the dotted lines in Figure 3a, these stained areas encompass both peri-ductal (light blue) and stromal (black) regions. The counts of these areas are reported in the graphs of Figure 3. In detail, the graph in Figure 3b measures α-syn immuno-stained ductal areas, given in μm^2^, while the graph in Figure 3c reports the increase in α-syn immuno-stained ductal area in PDAC tissue as a percentage of extra PDAC samples. The difference in the increase of staining counted in Figure 3b,c is due to a larger ductal area in PDAC compared with extra PDAC tissue. The percentage increase is attenuated due to a stained ductal area in PDAC tissue. In Figure 3d, the stromal α-syn immuno-stained areas intended as intense zones of a-syn immuno-staining according to patches and axon-like linear patterns are, again, in excess in PDAC compared with extra-PDAC tissue. The staining within stromal areas was irregular compared with the homogeneous staining in ductal cells.

### 2.4. Ultrastructural Morphometry and Stoichiometry Counts of α-Syn 

**Figure 4:**
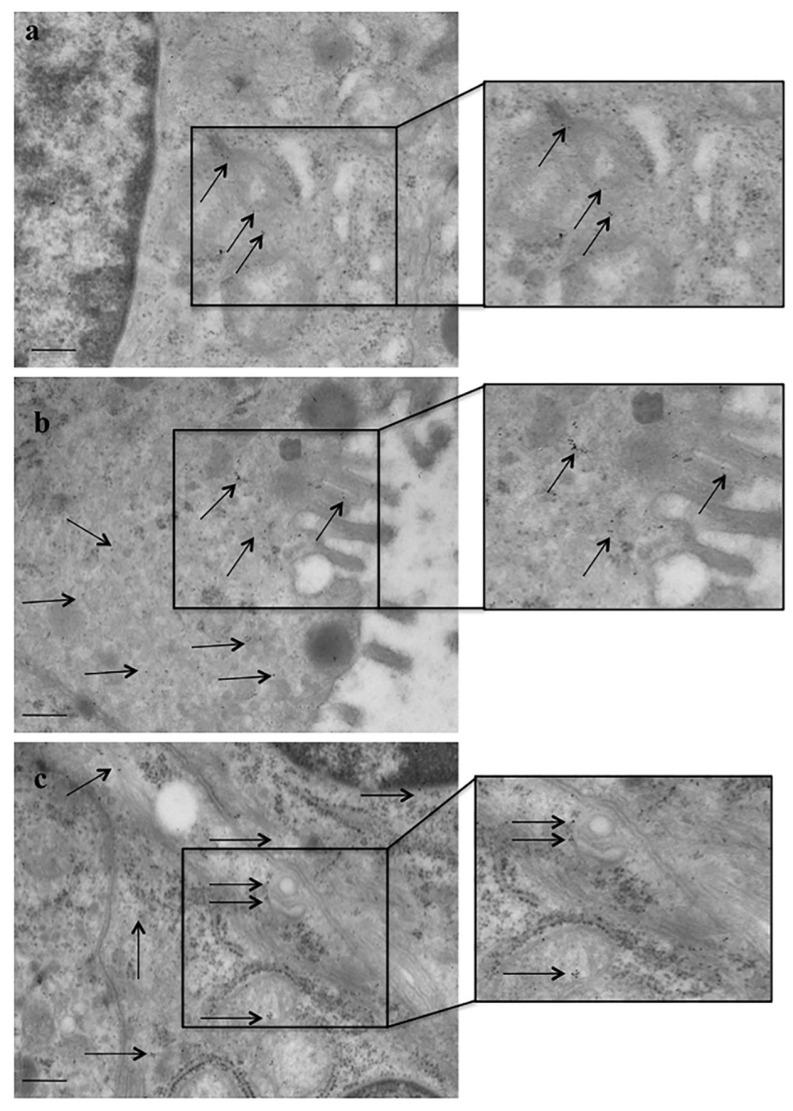
α-Syn immunocytochemistry in PDAC cells at TEM. Representative pictures show α-syn immuno-gold particles (arrows) within pancreatic ductal cells from extra PDAC (**a**) and PDAC (**b**,**c**) areas. Detection of α-syn was carried out for each single molecule. These counts allow stoichiometric quantitative measurement since each immuno-gold particles binds a single α-syn particle. The figures are representative images of how many immuno-gold particles are detectable in PDAC cells. The number of immuno-gold particles is counted both in cells from PDAC and extra-PDAC areas in order to build the graphs and to compare these measurements in Figure 5. Scale bar = 217 nm (low magnification); scale bar = 120 nm (inserts at high magnification).

**Figure 5:**
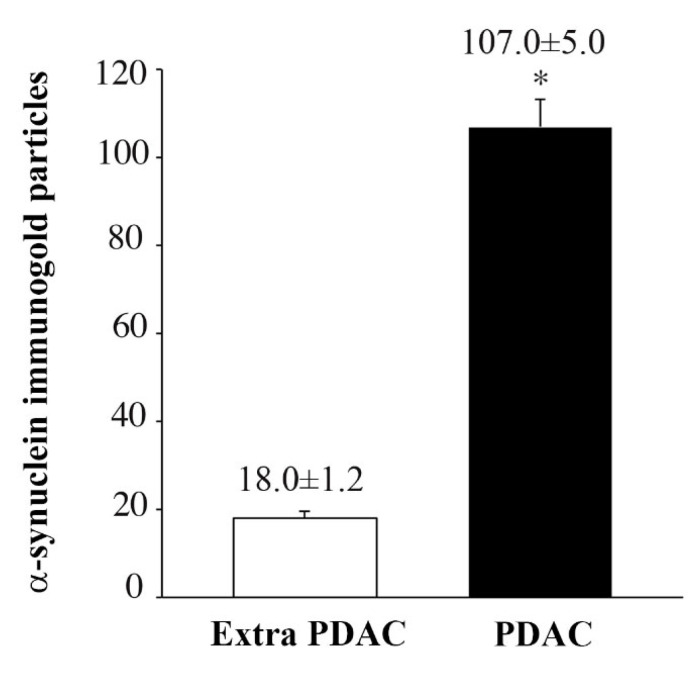
α-Syn immuno-gold particles per cell. Values are given as the mean ± S.E.M., from *N* = 60 cells per group. Immuno-gold particles are counted in single cells either from PDAC or extra-PDAC areas. The number are given as the mean + S.E.M. of 60 cells per group. Comparisons were made by using Student *t*-test. * *p* < 0.05.

Immunocytochemistry shows increased α-syn expression in PDAC ductal cells of all the patients, as shown in representative Figure 4. The detection of α-syn is based on the detection of single molecules due to immune-gold particles. These counts allow stoichiometric quantitative measurements since each immuno-gold particle binds a single α-syn particle. Figure 4 provides representative images of abundant immuno-gold particles detectable in PDAC cells. The number of immuno-gold particles was counted both in cells from PDAC and extra-PDAC areas in order to build the graphs and to compare these measurements in Figure 5.

The number of α-syn immuno-gold particles is massive in PDAC cells compared with extra-PDAC tissue, being five-fold in excess (107.0 ± 5.0 compared with 18.0 ± 1.2, respectively, (Figure 5). This immuno-electron microscopy again provides a striking quantitative difference between cells from PDAC compared with cells from extra PDAC areas.

### 2.5. Expression of α-Syn by Western Blotting

**Figure 6:**
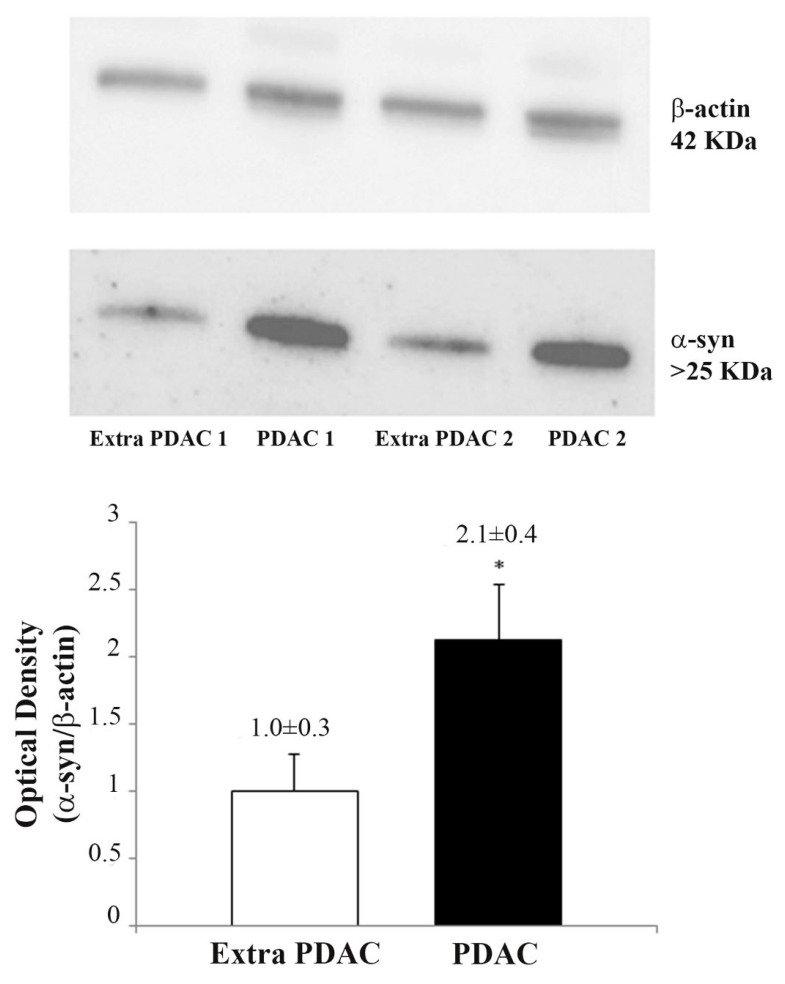
Representative Western blotting of α-syn. In the upper panels, four representative Western blots of a-syn and the house-keeping protein β-actin are reported for extra-PDAC and PDAC tissue. In the lower graph, relative optical density from *N* = 20 blots is reported. Values are given as the mean ± S.E.M., from *N* = 20 blots. Comparisons are made by using Student *t*-test. * *p* < 0.05.

Western blotting confirmed that α-syn was markedly expressed within samples punched from tumor pancreatic tissue (PDAC), while there was limited expression in non-cancer tissues (extra-PDAC), with a significant difference (2.1 ± 0.4 compared with 1.0 ± 0.3 optical density) (Figure 6). Although markedly different in PDAC compared with extra-PDAC tissue, these data are much less striking compared with those obtained with immuno-staining and ultrastructural morphometry; this is likely to depend on the in situ detection of α-syn within PDAC ductal cells (light and electron microscopy) compared with a blind sampling obtained by the gross dissection of the tumor (Western blotting). Nonetheless, the great amount of tissue sampled in this method compared with single cells or some ductal areas remains significantly different, and it allows a quick and feasible marker that still distinguishes the high amount of α-syn within PDAC.

### 2.6. Expression of α-Synuclein Significantly Increases When Perineural Invasion Occurs

**Figure 7:**
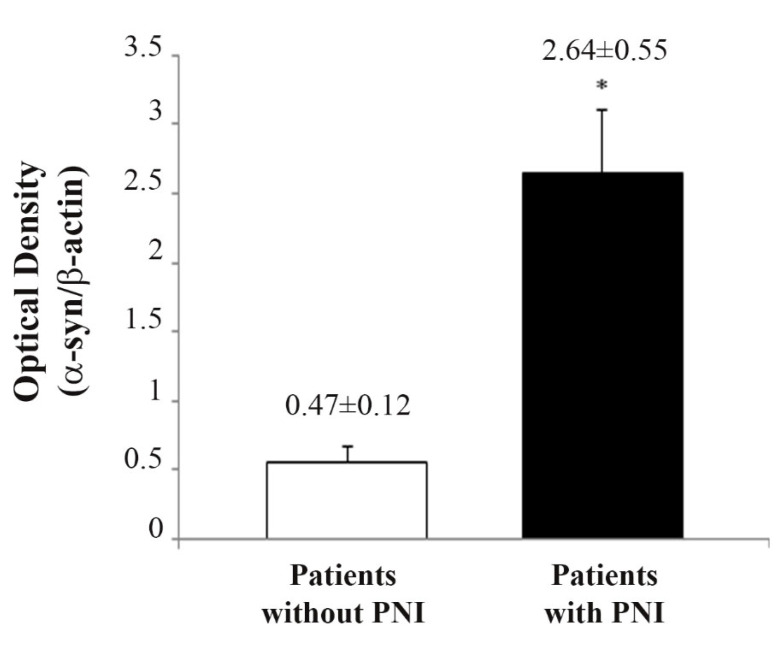
Comparison of α-synuclein expression between patients without PNI (*N* = 4) and patients with PNI (*N* = 16). α-syn immune-blot values are given as the mean ± S.E.M. Comparisons were made by using Student *t*-test. * *p* < 0.05.

α-Syn expression based on Western blotting and the occurrence of PNI in each patient is reported. In our group of patients, PNI was found in 16/20 patients (85%), while it was absent in 4/20 patients (15%). This was established at pathology for tumor staging by expressing the occurrence of PNI through the examination of peritumoral intrapancreatic nerves or extramural pancreatic nerves in the retroperitoneal connective tissue dorsal to the pancreas. When comparing the expression of α-syn between patients with and without PNI, a higher significant expression in the group with PNI according to Western blot analyses (2.64 ± 0.55 OD compared with 0.47 ± 0.12 OD, respectively, *p* = 0.035) was measured, as shown in Figure 7. Such a difference exceeds five-fold, and it is highly significant, which indicates that α-syn expression is much higher when perineural invasion takes place.

In the group of patients without PNI, all of them had G2 adenocarcinomas, without angioinvasion. In the PNI group, 13/16 patients (81.3%) had G2 adenocarcinomas, 3/16 (18.7%) G3 adenocarcinomas; moreover, 2/16 patients (12.5%) also had angioinvasion. When comparing the expression of α-synuclein between patients with the same grading (G2) and the absence of angioinvasion, with and without PNI (4 vs. 11 patients, respectively), it was still significantly higher in the group with PNI according to Western blot analyses (3.1 ± 0.76 OD vs. 0.47 ± 0.12 OD, respectively, *p* = 0.032). The large areas screened by Western blotting, although inadequate for expressing the specific amount of α-syn overexpression within specific compartments, still show a consistent increase in α-syn and appear to be useful for the quick screening of the tumor to correlate with neuroinvasion. 

## 3. Discussion

Pancreatic carcinogenesis is poorly understood. Various studies reveal that pancreatic carcinoma may feature multiple alterations of disease-related genes, including mutations and deletions of tumor-suppressant genes (such as p53, DPC4, p16, etc.), [50,51,52,53], as well as mutations and the overexpression of various oncogenes (such as K-ras, Her-2/neu) [54,55], which may extend to mismatch repair gene defects [56]. The identification and characterization of these genes increased our understanding of the molecular pathogenesis of pancreatic carcinoma. Among these genes, recent studies indicate that the manipulation of the autophagy-related pair signature predicts prognosis and immune activity in the course of PDAC [42]. These findings are in line with the occurrence of a high amount of autophagy-dependent substrates in PDAC, while triggering the search of specific autophagy-related molecules as markers or effectors in the natural course of PDAC. A number of autophagy-dependent substrates are under investigation. In fact, recent studies indicate an increased amount of the autophagy-dependent PrPc [43,44], which correlates with poor prognosis and neuro-invasion in patients affected by PDAC. The present study focuses on a specific autophagy-dependent substrate, the protein α-syn, which was only investigated in neurodegenerative disorders so far. In fact, an increase in α-syn is concomitant with autophagy failure in the course of specific degenerative disorders named synucleinopathies [57]. However, very recent findings indicate an increased amount of α-syn in neural crest-derived malignancies such as melanoma, where α-syn promotes cell proliferation and tumor spreading [25,26,58]. 

The present study indicates the marked overexpression of α-syn within PDAC in a small (*N* = 20) group of PDAC patients. The over-expression of α-syn occurs within PDAC-recruited areas, being very low within non-affected extra-PDAC surrounding tissue. The increase in α-syn occurs specifically at the level of affected ductal cells. In fact, by profiting off in situ detection of α-syn by light microscopy, it is possible to document the selective increase in the protein in all the perimeters of affected pancreatic ducts. Such an increase is very specific and spreads to all ductal cells, which leads to a marked (more than twentyfold) difference compared with non-affected tissue (the count of α-syn immuno-stained area in PDAC ductal regions exceeds more than ten-fold that counted in extra-PDAC control tissue (1340.1% ± 12.3% compared with 100.0% ± 14.4%, respectively). Similarly, within specific stromal areas, where patchy or linear axon-like α-syn immune-staining is detectable, the amount of α-syn is way in excess compared with non-affected tissue. In an effort to carefully quantify the over-expression of α-syn within PDAC tissue, we used stoichiometry detection. Since all cells in the affected ducts over-express α-syn, in these cells, the protein expression was quantified by measuring immuno-gold-identified single-protein molecules at ultrastructural stoichiometry. By using this procedure, the amount of α-syn protein exceeds, five-fold, the levels detected in extra-PDAC control tissue. Thus, both immuno-histochemistry and mostly ultrastructural morphometry are reliable procedure to assess the placement and amount of α-syn overexpression within specific cells and sites of PDAC tissue. However, following a routine quick sampling of a PDAC specimen, such a detailed count could be time-consuming, and the data provided need to be related to specific pancreas compartments. Thus, immuno-histochemistry and mostly ultrastructural stoichiometry of α-syn are very useful to detail the significance and placement of increased α-syn in PDAC. However, when gross information is required, a larger, less selective specimen needs to be assessed. The occurrence of a slight though significant increase in α-syn obtained by Western blotting is useful to obtaining a general sampling for α-syn levels within PDAC tissue. By using this procedure, the amount of increase is less pronounced since it includes non-specific compartments (such as the selective sampling of ductal cells); nonetheless, the difference between PDAC and extra-PDAC tissue is still marked and significant. By using Western blotting from PDAC tissue of all the patients, there was still the chance to detect a marked difference, which was related to the presence of perineural infiltration (PNI). In particular, PDAC tissue from patients with PNI has an α-syn amount exceeding five-fold that measured in PDAC tissues from patients without PNI. Thus, when considering the routine pathological assessment, we found that α-syn is related to the amount of peri-neural invasion, which represents a detrimental factor in the prognosis of PDAC. PNI represents a deleterious effect in the course of PDAC since it fosters cancer recurrence and reduces survival in PDAC patients [38]. Again, PNI was shown to be deleterious by the Ceyhan research team since it impairs prognosis and negatively impacts on the therapeutic response [36,37]. Thus, it is likely that the overexpression of α-syn enhances invasion and transmission through neural pathways of pancreatic carcinoma cells. In fact, recent studies indicate that α-syn may alter the cell phenotype and may promote cell growth [26,59,60,61,62,63,64,65]. Thus, it is not surprising that α-syn is abundantly expressed in PDAC. In this cancer, the concomitant overexpression of PrPc is described, which generates alpha-syn phosphorylation and aggregation [43,44]. It would be relevant to analyze whether α-syn occurring in PDAC also features Ser129 phosphorylation, as it occurs in melanoma cells, where this α-syn produces cell proliferation and is present in excess [65]. The silencing of α-syn in PDAC would answer a number of questions, which are fostered by the present data. The increase in α-syn is likely to depend on a dysfunction in its removal due to defective autophagy in PDAC.

Nonetheless, this is a vicious cycle since, in turn, α-syn overexpression inhibits the autophagy pathway through mTOR activation [66]. Thus, inhibiting mTOR would be another fascinating approach to question an alternative pathway to relent the course of PDAC. In fact, defective autophagy can be restored by mTOR inhibition [66,67,68]. Moreover, α-syn relays on the autophagy pathway for its clearance [69,70]. In line with this, the autophagy pathway is altered in PDAC [41,71]. Little is known about a potential cancerogenic role of α-syn, and no study is, to date, available for PDAC. To our knowledge, the present study is the first to report the over-expression of α-syn within PDAC samples from surgically resected patients. The abundant expression of α-syn is way in excess of controls, as shown by immuno-histochemistry and protein stoichiometry at electron microscopy. The gross results provided by Western blotting confirm such a significant increase. It is likely that, due to the random sampling of tissue harvested for Western blotting, a relevant amount of the sample contains necrotic and heterogeneous areas, which leads to potential antigen dilution, contributing to a flattening of the remarkable increase in α-syn. This is likely to depend also on the semi-quantitative approach of Western blots analysis and the non-linear regression between blots and protein content. Nonetheless, the increase in α-syn continues to be significant and correlates with PNI, making Western blotting of α-syn a promising approach as a biomarker for PNI in PDAC. Within a research scenario, in which the specific compartments where α-syn increases are analyzed to establish the biological significance, it is very likely that immuno-histochemistry provides the real status of α-syn over-expression in PDAC. Again, the authentic detection of single α-syn molecules by immuno-gold protein stoichiometry provides a reliable quantitative measurement, although this refers to micro-areas. Thus, the increase in α-syn in PDAC is valid with different methods, although the whole scenario provided by in situ antigen detection through light microscopy is likely to provide the best information concerning the whole intensity of the protein increase. This extends to the chance of detecting the stromal area where the antigens sometimes appear to be massively present along linear patterns, which are reminiscent of nervous plexus. These findings are quite a novelty in the PDAC literature as far as we know. In recent studies, we found that prion protein is highly expressed within PDAC [32,33].

The presence of α-syn within PDAC might be a marker that could contribute to understand the biology of the disease in terms of aggressiveness, explaining the uniquely preferred perineural invasion of this neoplasm, as suggested for PNI. This could be based on the peculiar neurotropism of α-syn and its abundant occurrence in neural tissue. Our preliminary results show a higher expression of α-syn in patients with PNI, which suggests a potentially higher aggressiveness and a relationship between α-syn and PDAC to represent the molecular basis of neurotropism. 

This study remains a preliminary work on surgically resected specimens of PDAC, and it represents the first step for a wider project in which hundreds and thousands of patients may be screened to confirm the sensitivity of α-syn as a disease marker. These studies should also consider the prognostic value of α-syn expression in PDAC tissue. The pool of our surgically resected patients will be followed-up in order to evaluate whether a significant correlation exists between α-syn expression and disease prognosis. The working hypothesis is that those patients with a high expression of α-syn may undergo a poor prognosis, with early relapse and a higher tumor spread, i.e., a higher neuro-tropism, a faster disease course, and a difficult response to therapeutic agents.

## 4. Materials and Methods

### 4.1. Patients and Specimens

Samples from tumors of patients surgically treated with pancreatic resections at the General Surgery Unit, University of Pisa were collected between January 2019 and June 2021. Written informed consent was obtained from patients to use their surgical specimens and clinical pathological data for research purposes. All patients had a preoperative suspicion of PDAC. Preoperative evaluation included medical history, physical, laboratory and radiological examinations, computed tomography (CT) and magnetic resonance imaging (MRI), often with magnetic resonance cholangiopancreatography (MRCP). In addition, abdominal ultrasound with and without contrast, endoscopic ultrasonography (EUS), and fine-needle aspiration (FNA) during EUS were also performed in selected patients. Preoperative data included age and gender. Pancreatic nodules not resulting in adenocarcinomas were ruled out from the study. Similarly, we could not proceed when tumor specimens were too small. When PDAC diagnosis was confirmed, the pathologist took specimens from the pancreatic tumor and from the non-tumoral pancreatic tissue adjacent to the lesion (extra-PDAC control). One specimen per group (extra-PDAC and PDAC) was fixed and kept in glutaraldehyde and paraformaldehyde for electron microscopy analysis; another one was rapidly frozen and kept at −80 °C for storage for Western blotting analyses (SDS-PAGE immunoblotting) to be carried out; a third couple was fixed and kept in formalin for immunohistochemistry. The histological data include the following: the histological type of the tumor, the grade of differentiation, the tumor size, the number of harvested lymph nodes, the number of metastatic lymph nodes, the presence of angio-invasion, and finally, peri-neural infiltration. 

Patients were staged after surgery according to the T and N definitions proposed for the AJCC 8th edition (pTNM), based on the pathology results. The proposed T-stage definitions are the following: T1 ≤ a cm maximal diameter, T2 > 2 ≤ c cm maximal diameter, T3 > 4 cm maximal diameter, T4 = locally unresectable. Extra-pancreatic extension was not included in T-stage definitions. The N-staging included the following: N0 = node negative, N1 = 1–3 nodes positive for metastatic disease, N2 ≥ c nodes positive for metastatic disease.

### 4.2. Immunohistochemistry

Morphological studies were carried out in control and PDAC tissues fixed in formalin 4%. For controls, we used non-affected neighboring tissue of the same patients (extra-PDAC tissue). After fixation, the samples were embedded in paraffin and 7–10 µm-thick tissue sections were cut and mounted on slides for hematoxylin and eosin (H & E) staining or immune-histochemical analysis. For H & E, the sections were plunged in the hematoxylin solution (Sigma, Aldrich, Milan, Italy) for 20 min, washed in running water, and then immersed in the Eosin solution (Sigma) for a few minutes. Finally, they were dehydrated in increasing alcohol solutions, clarified in xylene, and covered with DPX mounting medium (Sigma). For the immuno-histochemical experiments, the sections were first permeabilized by Triton X 0.1% for 15 min in TBS and then incubated in a blocking solution containing 10% normal goat serum (NGS) in TBS for 1 h at room temperature (RT). The sections were successively incubated with the α-syn antibody (Abcam, Cambridge, UK, Cat# ab27766, 1:150) overnight at 4 °C. After washing with BSA, the reaction with the Ab-I was revealed by using the secondary biotinylated antibody (Cat#BA9200). The immuno-peroxidase method was used to identify the complex: samples were incubated with avidin-biotin complex (DAB substrate Kit Peroxidase with nickel, Cat# SK-4100, Vector Laboratories, Burlingame, CA, USA) for 1 h at room temperature and stained with diamino-benzidine. The slides were mounted with the mounting medium DPX and were observed using the Nikon Eclipse 80i light microscope equipped with a digital camera connected to the NIS Elements software for image analysis (Nikon, Tokyo, Japan).

Quantification of the α-syn immunopositivity was carried out closely around the pancreatic ducts (periductal area) and within the stroma by using the software Image J. A measure of the periductal stained area out of the total periductal area was carried out in 100 ducts per group. Values are expressed both as mean percentage ± S.E.M. of the positive peritubular area (not tumoral tissue = 100) and mean positive area ± S.E.M. per group.

Measure of the α-syn immunopositivity within the stroma was carried out in 20 sections per group. Values are given as the mean ± S.E.M. of the α-syn immune-stained area. 

### 4.3. SDS-PAGE Immunoblotting

Pancreatic tissue was lysed in ice-cold lysis buffer (50 mM Tris-HCl, pH 7.5, 150 mM NaCl, 5 mM EDTA, 1% SDS, 0.1% IGEPAL) containing Complete Protease Inhibitor Cocktail Tablet (Santa Cruz Biotechnology, sc-29130, Dallas, TX, USA). Then, tissues were sonicated and homogenized, and then, they were centrifuged at 5000× *g* for 5 min at 4 °C. The supernatant was collected, and the protein concentration was determined using a protein assay kit (Sigma-Aldrich, Cat# TP0300,). Samples (30 μg) were electrophoresed on 4–20% sodium dodecyl sulfate-polyacrylamide gel (Cat#4568093, Bio-Rad Laboratories). Following electrophoresis, proteins were transferred to a nitrocellulose membrane (Cat#1704158, Bio-Rad Laboratories, Segrate, Italy). The membrane was immersed in blocking solution containing PBS with 0.05% Tween-20 (PBS-T) and 5% non-fat dried milk (Sigma-Aldrich, Cat# 70166), for 2 h at room temperature on a plate shaker. Subsequently, the membrane was incubated overnight at 4 °C with primary antibody anti-α-syn (1:800, Cat #ab27766, Abcam) diluted in PBS-T containing 2.5% non-fat dried milk (Sigma). The blots were washed three times with PBS-T and incubated for 1 h with goat anti-mouse horseradish peroxidase-labeled secondary antibody (1:3000; Cat# 04-18-06, KPL *Antibodies & Conjugates*, SeraCare, Kampenhout, Belgium) diluted in PBS-T containing 2.5% non-fat dried milk (Sigma). The bands were visualized with enhanced chemiluminescence reagents (Clarity Western ECL Substrate; Cat#1705061, Bio-Rad Laboratories) and image analysis was carried out by ChemiDoc MP (Bio-Rad Laboratories). The intensity of the blotting was measured using the ImageJ software and was normalized for the related housekeeping protein (β-actin Abcam, Cambridge, Cat# ab8227). Values are expressed as the mean ± S.E.M. of the optical density (OD). Western blots of PDAC tissues were compared with control tissues. 

### 4.4. Electron Microscopy

For electron microscopy, small fragments of normal and tumoral pancreatic tissue were fixed in 0.1% glutaraldehyde and 2% paraformaldehyde phosphate buffer pH 7.4 for 90 min. It used a fixing solution, minimally covering the antigen epitope while fairly preserving the tissue architecture. After washing in buffer, samples were post-fixed in 1% OsO4-buffered solution for 1 h at 4 °C. The samples were then dehydrated in a series of increasing ethanol concentration (50%, 70%, 90%, 95%, 100%) followed by propylene oxide for 20 min. Afterward, samples were embedded in a mixture of Epon Araldite and propylene oxide (ratio of 1:1 overnight at room temperature), and finally, they were embedded in pure EponAraldite resin for 72 h at 60 °C. Ultrathin sections were stained with uranyl acetate and lead citrate and examined with a Jeol Jem 100SX transmission electron microscope (TEM) (Jeol, Tokyo, Japan) at an acceleration voltage of 80 kV.

### 4.5. Post-Embedding Immunocytochemistry

A post-embedding procedure was carried out on ultrathin sections collected on nickel grids. Grids were washed in PBS and incubated in a blocking solution containing 10% goat serum and 0.2% saponin for 20 min, at room temperature, then they were incubated with a primary antibody solution containing: anti-mouse α-synuclein antibody (Abcam, Cat# ab27766 diluted 1:50), 0.2% saponin and 1% goat serum in a humidified chamber overnight, at 4 °C. After washing in PBS, grids were then incubated with anti-mouse secondary antibodies conjugated with gold particles (10 nm mean diameter, BB International Crumlin, UK, Cat# EM.GMHL10/0.25), which were diluted 1:40 in PBS containing 0.2% saponin and 1% goat antiserum for 1 h at room temperature. Control sections were incubated with secondary antibody only. After washing in PBS, grids were incubated on a droplet of 1% glutaraldehyde for 3 min; an additional extensive washing of grids with distilled water was carried out to remove excess salt traces. Sections were stained with uranyl acetate and lead citrate and examined at TEM. For each experimental group (extra PDAC and PDAC), 12 grids were observed for a total of 60 cells, which were selected from the region in which the cellular ducts were present. In order to measure the expression of the immuno-gold particles, we counted the total number of gold particles in each cell (*N* = 60) examined; TEM analysis was performed at a magnification of 8000×, which allowed the concomitant visualization of immuno-gold particles and all cell organelles. The expression of α-syn was revealed by counting the immuno-gold particles, both in control and PDAC groups. 

### 4.6. Statistical Analysis

#### 4.6.1. Tissue Sampling

As reported, the area selected as PDAC was sampled at a distance of at least 1 cm from the area sampled as extra-PDAC. In addition, for the sake of correct tissue sampling we rule out concomitant pancreatic pathology. In fact, a few patients selected for the study show focal pre-neoplastic lesions (particularly PanINs) and/or some areas affected by chronic pancreatitis; in the present study, tissue sampling used as extra-PDAC tissue was obtained from pancreatic parenchyma far from the neoplasm (at least 1 cm away). Moreover, the sampled tissue was histologically examined as shown in Figure 1 to assess the absence of pre-neoplastic lesions or incipient chronic pancreatitis. In order to provide a view of the border of PDAC tissue, Supplementary Figure S1 provides a direct visualization of the tissue, which immediately surrounds the PDAC area. In any case, one should consider that sampled tissue for extra-PDAC regions used to quantify the amount of α-syn was distant at least 1 cm from the PDAC area.

#### 4.6.2. Homogeneity of Specimen/Tumor Cellularity

The cellularity of pancreatic tissue is by definition highly heterogeneous. This point was dealt with in discussing the difference for the occurrence of α-syn throughout various procedures. In fact, the presence of ductal regions (fully positive for α-syn) or scattered α-syn densely immuno-stained area is dispersed, and it is scattered when a non-specific piece of tissue is used for Western blotting (WB). This is why WB provides less marked increase in α-syn within PDAC areas compared with immuno-staining and immuno-gold stoichiometry. In fact, these latter procedures are aimed to detect in situ those spots where α-syn specifically increases in PDAC. These latter methods better define the quantity and measurements of the increase in α-syn. Therefore, they are used to quantify the amount of the protein in the present study.

In contrast, the use of a quick/low cost procedure such as WB is used here to assess routinely whether, considering even heterogeneous cellularity of a gross specimen of PDAC/extra PDAC tissue, may keep the difference in α-syn expression as significant. Thus, heterogeneous cellularity inherent to samples used for Western blotting represents a point, which would strengthen the finding of α-syn over-expression. In keeping with this, even when non-specific regions are blotted the overall increase in the protein should remain significant. This is why Western blotting is used as a routine procedure to assess α-syn in the groups of PNI and non PNI patients. This is aimed to provide a routine screening. One should consider that this routine measurement does not rule out the importance of a careful cell-based analysis of α-syn in PNI compared with extra-PNI areas by counting selectively the ductal areas. This is even more selective when stoichiometry-measured α-syn molecules are counted within ductal cells. In fact, electron microscopy of ductal cells, used in the present study to carefully quantify the occurrence of α-syn is based on in situ stoichiometry of α-syn molecules in specific areas (for instance ductal cells). These measurements provide the gold-standard for quantifying α-syn within PDAC compared with extra PDAC areas. Instead, routine screening to test whether a significance is kept even at Western blotting to distinguish patients with PNI and without PNI was carried out on Western blotting of all PDAC from 20 patients as reported in Supplementary Figure S2.

#### 4.6.3. Semi-Quantitative and Quantitative Measurements

Western blotting is not a sophisticated approach of analytical chemistry, and it does not represent an absolute quantitative technique. It is rather a semi-quantitative assay. A careful cell-based analysis of α-syn is important for the present study. Therefore, we counted the number of α-syn molecules specifically within ductal cells. This was carried out by using ultrastructural stoichiometry at transmission electron microscopy. In fact, this method provides the gold standard for in situ quantitative counts of single antigenic molecule with a ratio of 1:1 between α-syn protein and immuno-gold particle. 

In fact, Western blotting does not provide protein absolute amount (results from western blotting never express a known amount of assayed protein molecules). It would be inappropriate to use Western blotting for quantitative analysis and even to draw regression analysis of protein content which requires mass spectrometry or in situ can be done only by TEM immuno-gold (as carried out in the present study).

#### 4.6.4. The Statistical Issue of Peri-Neural Invasion (PNI) 

Perineural invasion (PNI) is a prominent feature in pancreatic cancer. In classic staging it is expressed as either absent of present. Therefore, patients are defined either as owning PNI or not. In each patient, α-syn occurring within PDAC tissue is blotted and the mean + S.E.M. of the blots of α-syn in PDAC patients with PNI were compared with the mean + S.E.M of the blots of α-syn in PDAC patients without PNI. The inferential statistic considering the mean, the variation (here expressed by S.E.M) is aimed to assess whether a significant difference could be detected by the Student’s t-test (two groups: PNI and non-PNI, and one measurement: the amount of α-syn). In this way a semi-quantitative assessment of the amount of α-syn in PDAC patients depending on the presence of PNI is established and compared.

The occurrence of PNI is defined in common pathology as present or non-present. In the present study, based on current pathology assessment, there is no purpose to quantify the amount of PNI. The Western blotting for the amount of α-syn represents the semi-quantitative assessment in two groups of patients (PNI and non-PNI).

Occurrence of PNI is established according to routine scoring used in tumor staging at pathology exam.

In fact, current pathology established PNI in PDAC as a non-quantifiable event; routinely PNI is described only dichotomously (either “present” or “absent”). This is substantiated by pathology literature analyzing PNI in PDAC patients, which confirms that no quantitative assessment is presently uniformly established for PNI in pancreatic surgical specimen [72,73,74].

The effort to express a quantitative assessment of PNI is a matter of intense pathological efforts. In fact, PNI is a crucial contributor to an increased risk of pancreatic cancer mortality and extreme pain that occurs when cancer progresses [75], some authors tried to investigate a sort of “amount of PNI” by expressing intra- and extra-pancreatic perineural invasion [76]. 

However current data do not converge and they rather express discordant issues. This is why the presence vs. the absence of PNI is currently used to classify PDAC patients. 

In summary, in pancreatic cancer PNI is assessed qualitatively as “present” or “absent” simply based on extensive light microscopy. PNI is present when at least just one nerve may be involved by the neoplasm. This is enough for a qualitative definition of PNI as “present”.

#### 4.6.5. Overall Analysis

Continuous variables with normal distribution are expressed as mean ± standard error of the mean (S.E.M.) and compared using unpaired two-tailed Student *t*-test (since comparisons involved two unpaired groups of patients or samples, and one measurement: the amount of α-syn). 

We always used S.E.M. in all the graphs, when comparing the expression of α-syn within PDAC vs. extra-PDAC tissue or when comparing the expression of α-syn in PDAC patients with PNI with PDAC patients without PNI. 

When *p* < 0.05, the H_0_ hypothesis was rejected. The statistical analysis was performed using SPSS (Statistical Production and Service Solution for Windows, SPSS Inc., Chicago, IL, USA), version 23.

## 5. Conclusions

These results indicate that α-syn is markedly over-expressed within cells of PDAC tissues compared with extra-PDAC controls. The over-expression of α-syn significantly occurs in the presence of perineural invasion as assessed at pathological staging; this could be related to the role of α-syn in enhancing the emergence of a more aggressive behavior of cancer in PDAC cells, promoting their aggressiveness and neurotropism. These data provide a step forward in our understanding of PDAC biology. In fact, α-syn may represent a marker of disease severity in PDAC. Further studies with a higher number of patients are in progress to validate these results and to investigate potential clinical applications. The dissemination of these significant early findings should help to establish the clinical significance of α-syn in PDAC in a large cohort of patients.

## Figures and Tables

**Table 1:** Post-operative data according to pathology results.

Number of Patients, *n*	20
Pancreatic ductal adenocarcinoma, *n* (%)	20 (100%)
Mean tumor dimension, cm	3.4 ± 0.3 (1.5–6.5)
Mean harvest lymph nodes, *n*	32.1 ± 3.3 (14–62)
Mean metastatic lymph nodes, *n*	4.9 ± 0.8 (1–12)
Angioinvasion, *n* (%)	2 (10%)
Perineural infiltration (PNI), *n* (%)	16 (80%)
	Grading, *n* (%)
G2	17 (85%)
G3	3 (15%)
	T status, *n* (%)
T1	1 (5%)
T2	11 (55%)
T3	8 (40%)
	N status, *n* (%)
N0	2 (10%)
N1	5 (25%)
N2	13 (65%)
Stage, *n* (%)	
I	2 (10%)
II	6 (30%)
III	12 (60%)
Stage of patients without PNI, *n* (%)	
III	4 (100%)
Stage of patients with PNI, *n* (%)	
I	2 (12.5%)
II	6 (37.5%)
III	8 (50%)
Grading of patients without PNI, *n* (%)	
G2	4 (100%)
Grading of patients with PNI, *n* (%)	
G2	13 (81.3%)
G3	3 (18.7%)

Three cancer-stage groups were identified according to pTNM (AJCC 8th edition); stage I (*n* = 2, 10%), stage II (*n* = 6, 30%), and stage III (*n* = 12, 60%).

## Data Availability

The data that support the findings of this study are available from the corresponding author upon reasonable request.

## Supplementary Materials

The following supporting information can be downloaded at: https://www.mdpi.com/article/10.3390/ijms23073775/s1
