# Supplementary material for: In Pancreatic Adenocarcinoma Alpha-Synuclein Increases and Marks Peri-Neural Infiltration
Source: Int J Mol Sci. 2022 Mar 29;23(7):3775. doi: 10.3390/ijms23073775 (PMC8999122; doi:10.3390/ijms23073775)
Supplement: Supplementary file 1 [file ijms-23-03775-s001.zip › ijms-1591731-supplementary.pdf]

International Journal of
Molecular Sciences

MDPI

Supplementary Materials

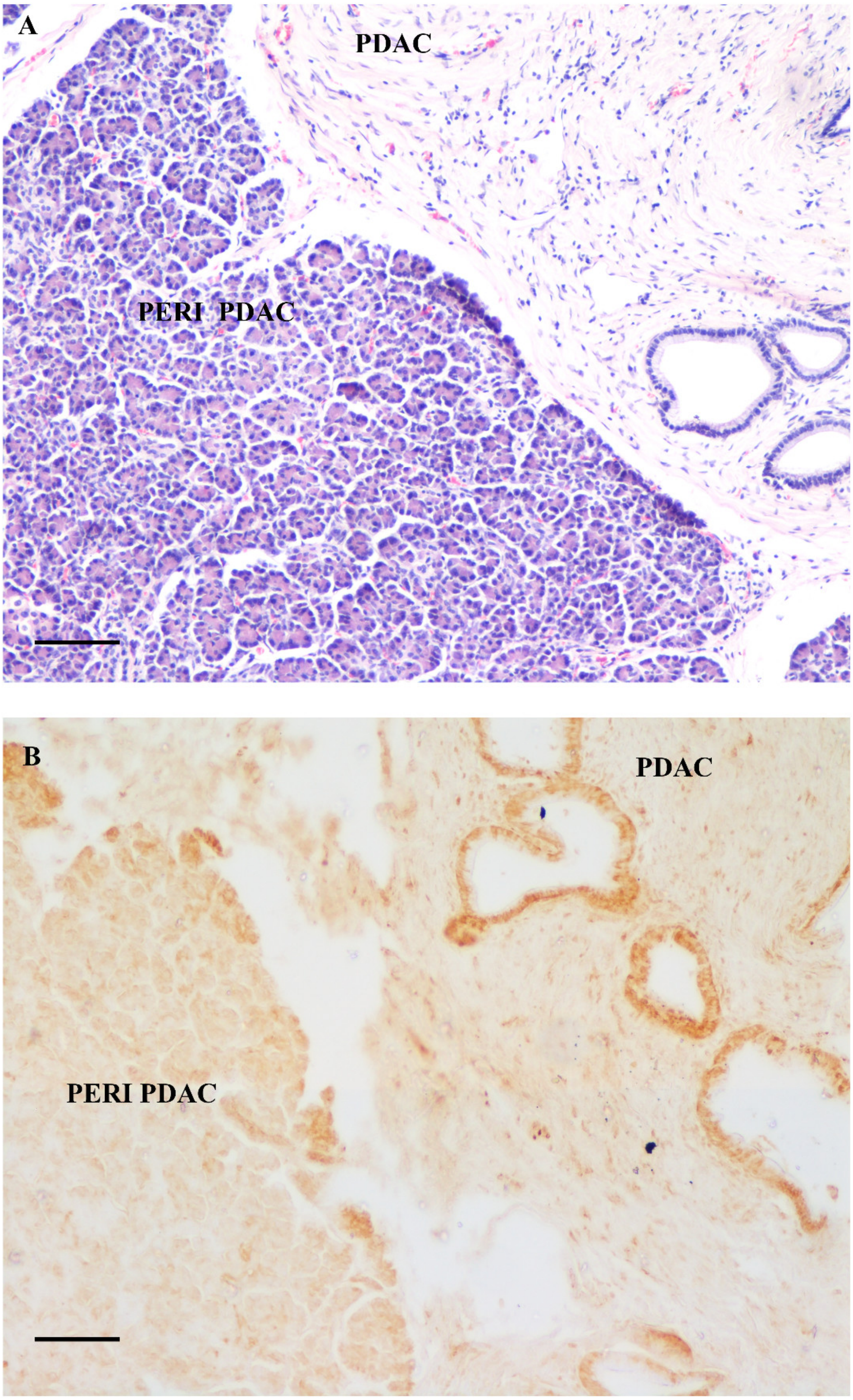

Supplementary Figure S1. Two whipple resections showing normal pancreatic tissue at the edge with PDAC. (**A**) Hematoxylin and Eosin- and (**B**) α-syn immunoperoxidase-stained tissue are

shown. In both pictures, the normal architecture of pancreatic tissue (PERI PDAC) is evident on the left, while PDAC tissue is shown on the right. Scale bar=50 μm.

A

α-syn β-actin

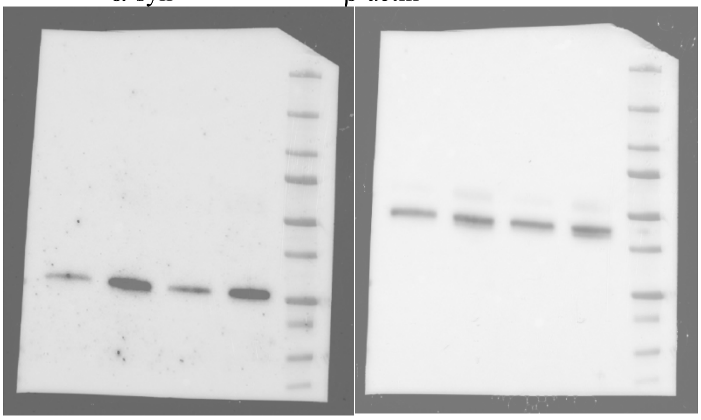

A a B b A a B b

Western blot α-syn and β-actin :

A extra PDAC patient 1

a PDAC patient 1

B extra PDAC patient 2

b PDAC patient 2

B

α-syn β-actin

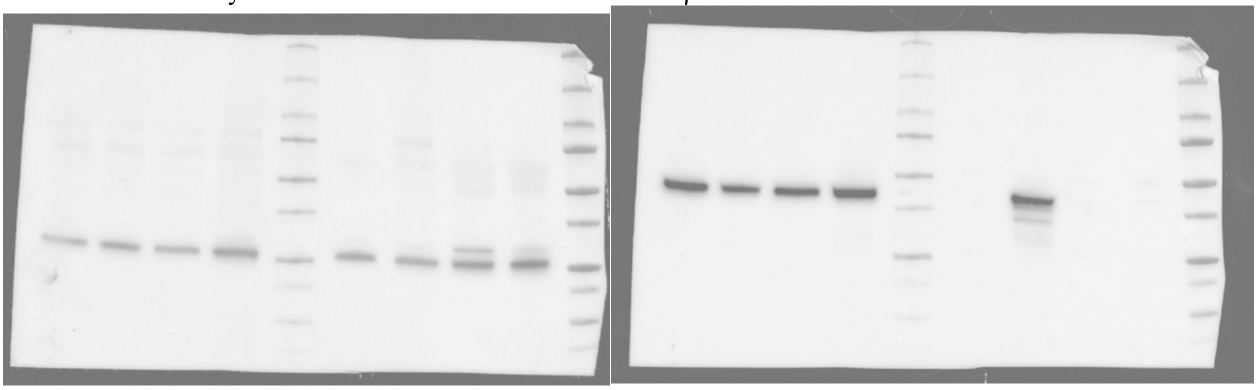

C c D d E e C c D d E e

Western blot α-syn and β-actin :

C extra PDAC patient 3

c PDAC patient 3

D extra PDAC patient 4

d PDAC patient 4

E extra PDAC patient 5

e PDAC patient 5

C

α-syn β-actin

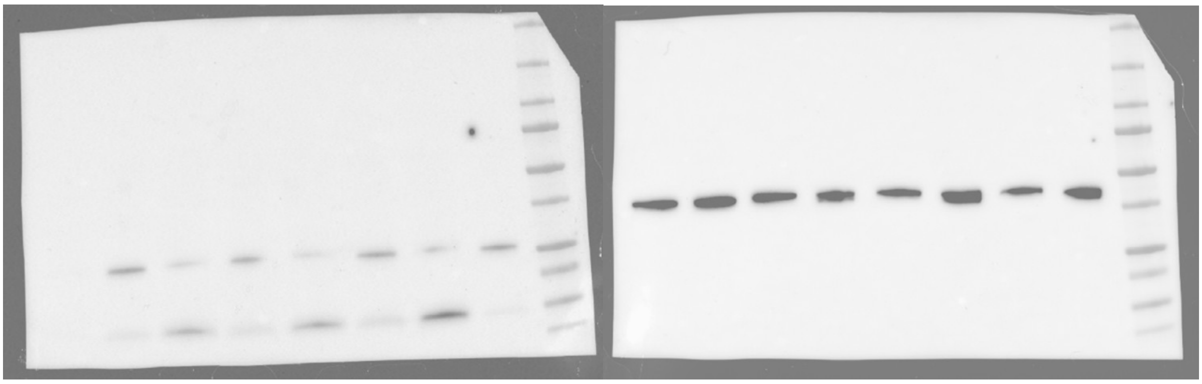

F f G g H h I i F f G g
H h I i

Western blot α-syn and β-actin :
F extra PDAC patient 6
f PDAC patient 6
G extra PDAC patient 7
g PDAC patient 7
H extra PDAC patient 8
H PDAC patient 8
I extra PDAC patient 9
i PDAC patient 9

D

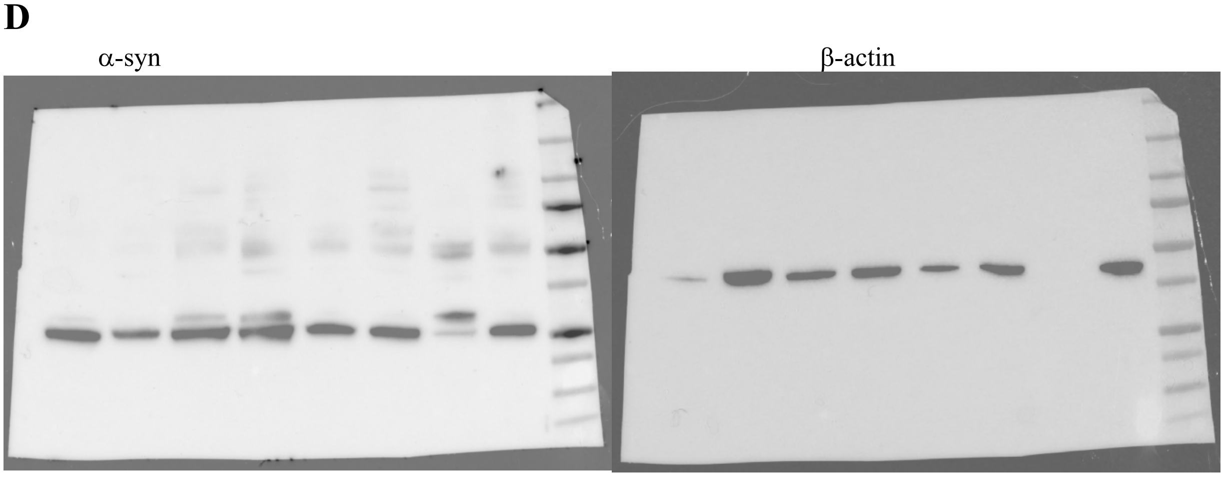

J j K k L l J j
K k L l

Western blot α-syn and β-actin :
J extra PDAC patient 10
j PDAC patient 10
K extra PDAC patient 11
k PDAC patient 11
L extra PDAC patient 12
l PDAC patient 12

E

α-syn β-actin

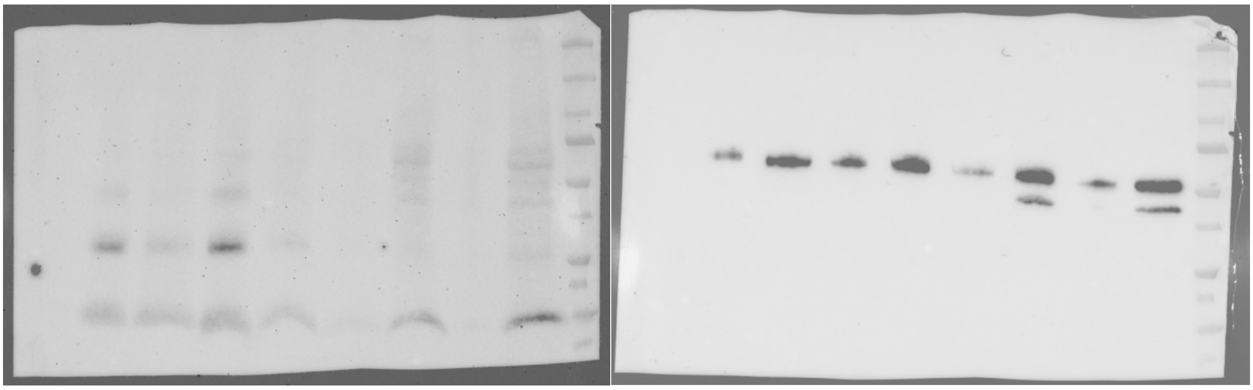

M m N n O o P p M m N
n O o P p

Western blot α-syn and β-actin :
M extra PDAC patient 13
m PDAC patient 13
N extra PDAC patient 14
n PDAC patient 14
O extra PDAC patient 15
o PDAC patient 15
P extra PDAC patient 16
p PDAC patient 16

F

α-syn β-actin

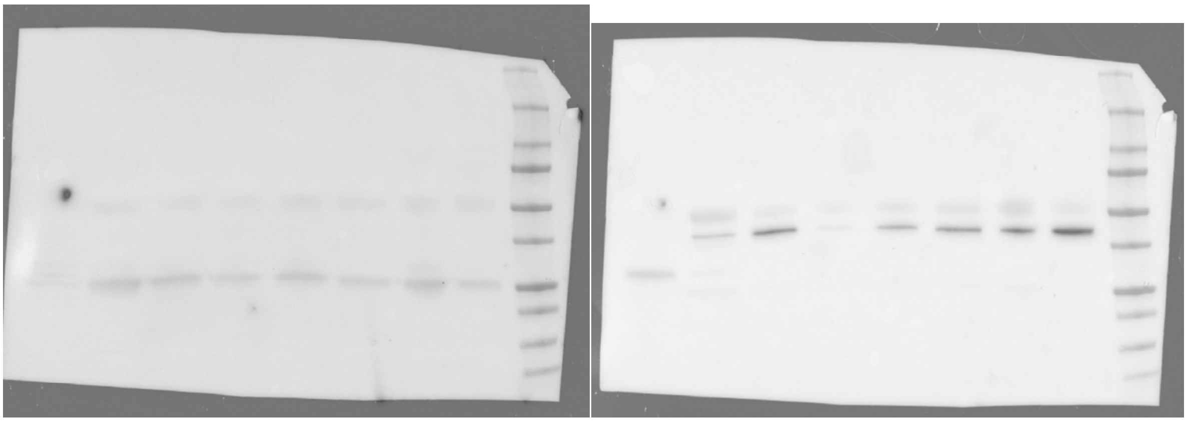

Q q R r S s T t Q q R r S s T t

Western blot α-syn and β-actin:

Q extra PDAC patient 17
q PDAC patient 17
R extra PDAC patient 18
r PDAC patient 18
S extra PDAC patient 19
s PDAC patient 19
T extra PDAC patient 20
t PDAC patient 20

Supplementary Figure S2. Western blots for α-syn form patients 1 and 2 (**A**), patients 3, 4 and 5 (**B**), patients 6, 7, 8 and 9 (**C**), patients 10, 11 and 12 (**D**), patients 13, 14, 15 and 16 (**E**) and patients 17, 18, 19, and 20 (**F**).
